# Circadian regulation of liver metabolism: experimental approaches in human, rodent, and cellular models

**DOI:** 10.1152/ajpcell.00551.2022

**Published:** 2023-08-29

**Authors:** Lorna J. Daniels, Danielle Kay, Thomas Marjot, Leanne Hodson, David W. Ray

**Affiliations:** ^1^Oxford Centre for Diabetes, Endocrinology and Metabolism, Radcliffe Department of Medicine, University of Oxford, Oxford, United Kingdom; ^2^NIHR Oxford Biomedical Research Centre, John Radcliffe Hospital, Oxford, United Kingdom; ^3^Kavli Centre for Nanoscience Discovery, University of Oxford, Oxford, United Kingdom

**Keywords:** circadian, glucose, lipids, misalignment, shift work

## Abstract

Circadian rhythms are endogenous oscillations with approximately a 24-h period that allow organisms to anticipate the change between day and night. Disruptions that desynchronize or misalign circadian rhythms are associated with an increased risk of cardiometabolic disease. This review focuses on the liver circadian clock as relevant to the risk of developing metabolic diseases including nonalcoholic fatty liver disease (NAFLD), insulin resistance, and type 2 diabetes (T2D). Many liver functions exhibit rhythmicity. Approximately 40% of the hepatic transcriptome exhibits 24-h rhythms, along with rhythms in protein levels, posttranslational modification, and various metabolites. The liver circadian clock is critical for maintaining glucose and lipid homeostasis. Most of the attention in the metabolic field has been directed toward diet, exercise, and rather little to modifiable risks due to circadian misalignment or disruption. Therefore, the aim of this review is to systematically analyze the various approaches that study liver circadian pathways, targeting metabolic liver diseases, such as diabetes, nonalcoholic fatty liver disease, using human, rodent, and cell biology models.

**NEW & NOTEWORTHY** Over the past decade, there has been an increased interest in understanding the intricate relationship between circadian rhythm and liver metabolism. In this review, we have systematically searched the literature to analyze the various experimental approaches utilizing human, rodent, and in vitro cellular approaches to dissect the link between liver circadian rhythms and metabolic disease.

## Graphical abstract

**Figure:**
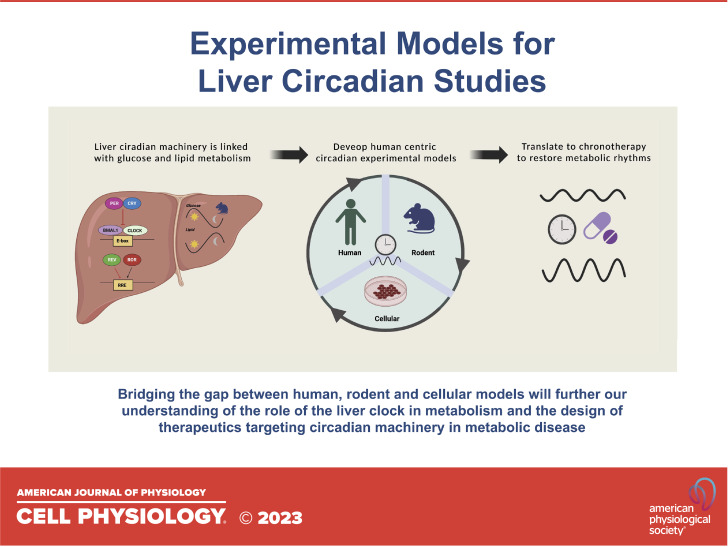

## CIRCADIAN SYSTEM

**Figure 1.**
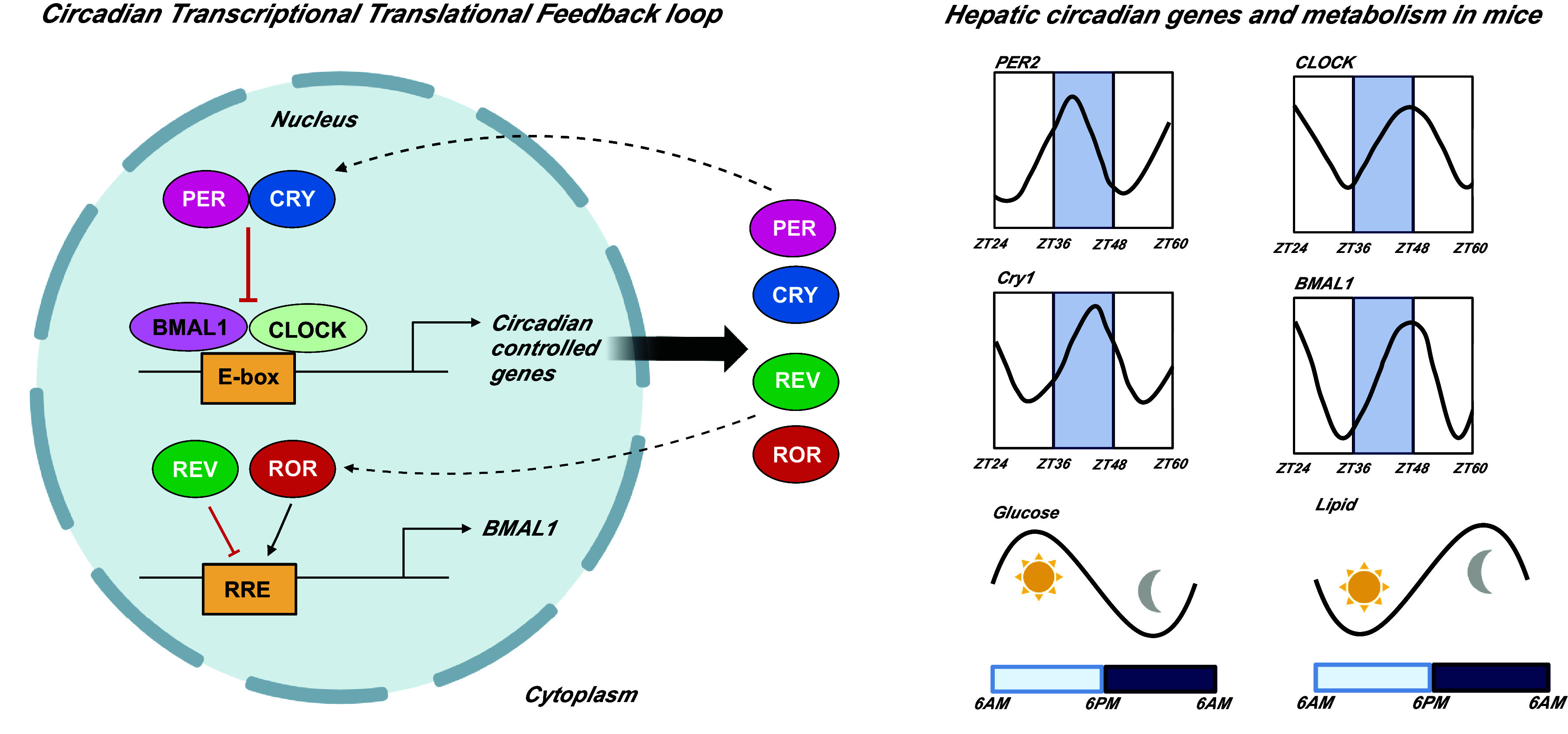
Circadian transcriptional translational feedback loop and hepatic circadian genes and metabolism in mice. Traces for circadian genes adapted from circaDB database. *BMAL1*, Arnt-like protein; *CLOCK*, circadian locomotor output cycles kaput; *CRY*, *Cryptochrome 1/2*; *PER*, *Period 1/2/3;* ZT, Zeitgeber time. Figure created using Biorender.com.

Circadian rhythms are endogenous oscillations with a period of ∼24 h. Such oscillations enable organisms to anticipate changes in the environment, and to tune responses by time of day. In mammals, the master circadian clock is located in the suprachiasmatic nucleus (SCN) in the hypothalamus, which is responsive to light signals transmitted from the retina by direct neural projections. The SCN master clock entrains clocks in peripheral tissues via autonomic and endocrine signals (1). At the molecular level, the clock consists of a transcription-translational feedback loop, with a periodicity of ∼24 h (2). The positive loop consists of core clock genes, circadian locomotor output cycles kaput (*CLOCK*), and Arnt-like protein (*BMAL1*) (Fig. 1) (3, 4). There are two negative loops, one is made up of *Period 1/2/3(PER*) and *Cryptochrome 1/2 (CRY)* genes (5, 6), and the other by the *REVERB/ROR* gene products that trans repress or trans activate the core clock gene *BMAL1*, respectively. This feedback loop operates to regulate a number of physiological processes including heart rate, hormone, and glucose levels (7, 8). Peripheral clocks can also be affected by other Zeitgebers (time cues) such as exercise and feeding. Disruptions that desynchronize or misalign circadian rhythms are linked with cardiometabolic disease (9, 10).

### Liver Circadian Biology

In this review, we will focus on the liver circadian clock. Approximately 40% of the hepatic transcriptome oscillates with circadian characteristics (11), and in addition there are 24-h rhythms in protein abundance, posttranslational modification, and many metabolites also oscillate (12). The liver clock responds strongly to the timing of food intake (13–15), and the liver transcriptome is enriched with transcripts involved in cellular adaptation to nutrients (16). Therefore, there is strong evidence of a link between the liver clock machinery, and energy metabolism. Studies in both humans and rodents taken through a circadian challenge (alteration to sleep and feeding behavior) support an association between circadian misalignment and risk factors for, or the prevalence of, energy metabolic diseases including nonalcoholic fatty liver disease (NAFLD), insulin resistance, and type 2 diabetes (T2D).

#### Circadian regulation of liver glucose signaling.

The liver has a crucial role in regulating glucose homeostasis, and these regulatory circuits are also affected by circadian phase. This allows anticipation and tuned responses to variations in glucose supply and demand during the fed and fasted states (17). In the active phase (day for human, night for mouse), following consumption of a mixed meal, blood glucose rises, and the liver takes up glucose and utilizes it for glycolysis, or stores it as glycogen. In the rest, or fasting, phase the liver adapts to the change in energetic demand by increasing gluconeogenesis and glycogenolysis (18). Key rhythmic factors that play a role in these processes include: glucose transporter 2 (GLUT2), glycogen synthase (GYS), glycogen synthase kinase (GSK3), and the insulin receptor (InsR) (19, 20).

The rhythmic nature of liver glucose metabolism predicts that alterations to the liver clock have implications for glucose homeostasis. For example, genetic disruption of the circadian gene *Bmal1* in mice resulted in a reduction in the liver glucose transporter (Glut2), leading to a decrease in postfeeding glucose uptake in mutant mice compared with wild-type mice (20). Cryptochromes (circadian gene) have also been associated with regulation of hepatic gluconeogenesis, affecting G protein-coupled receptor-dependent cAMP accumulation, and activation of cAMP response element-binding protein (CREB) (21). Overexpression of Cry1, specifically in the liver, resulted in lower blood glucose levels and increased insulin sensitivity in diabetic mice (22). Cryptochromes have also been found to serve as promiscuous repressors of nuclear receptor transactivation, notably the fasting-activated glucocorticoid receptor (GR), and so inhibiting fasting induction of phosphoenolpyruvate carboxykinase, a key gluconeogenic enzyme encoding gene (23, 24).

#### Circadian regulation of liver lipid signaling.

The hepatic circadian clock powerfully regulates lipid metabolism. Studies utilizing Per2, Clock, and REV-ERBα/β knockout models have revealed alterations to the diurnal levels of plasma levels of free fatty acids (also known as nonesterified fatty acids, NEFAs), triglycerides (TG), and cholesterol (25–27); the hepatic circadian machinery has been linked with critical steps of lipid metabolism. Genes involved in triglyceride (TG) synthesis (*Gpat2, Agpat1/2, Lipin1/2*, and *Dgat2*) are influenced by the circadian clock leading to a peak and trough of hepatic TG levels (in mice) during the rest and active phases, respectively (28). Hepatic circadian genes also participate in *1*) fatty acid synthesis by controlling the expression of *Elvol3, Elovl6, Fas* (29), *2*) regulating β oxidation and ketone body production (30), and *3*) determining the expression of key lipid responses nuclear receptors (NRs), liver X receptors (LXRs) peroxisome proliferator-activated receptor (PPAR) (29, 31). The role of the circadian machinery in lipid metabolism has led to it being linked with hepatic steatosis, and has been extensively described elsewhere (32, 33). For a comprehensive overview of circadian genetic mouse studies investigating glucose and lipid signaling, see the study by Kalsbeek et al. (21).

### Circadian Experimental Models: Human, Rodent, and Cellular

Most of the attention in the metabolic field has been directed toward diet, exercise, and rather little to modifiable risks due to circadian misalignment or disruption. Circadian misalignment is common in modern industrialized societies where artificial light exposure coupled with work and social demands often leads to environment and behavior running out of alignment with the internal, or circadian time (34). In fact, it is estimated that 20% of the working population is made up of shift workers, 33% of the population sleep less than 6 h per night (35), and 69% experience social jet lag (36). Therefore, developing a deeper understanding of circadian biology and how it relates to the regulation of hepatic glucose and lipid metabolism could have significant implications for identifying novel therapeutic approaches for metabolic disease.

The aim of this review was to reconcile why there are discrepant findings between studies investigating circadian regulation of metabolism. To do this we have brought together information from studies that have utilized human, rodent, and cellular experimental approaches. A comprehensive review of the literature was carried out in April to July 2021 via the electronic database PubMed. The key questions asked were as follows: *Human*: *1*) What are the effects of shiftwork on fasting glucose tolerance and insulin resistance? *2*) What are the effects of experimental misalignment protocols in healthy subjects on fasting tolerance and insulin resistance? *Rodent (hepatic clock)*: *1*) What are the effects of a diabetic intervention in a rodent model on hepatic regulation of rhythmicity of core clock genes (CLOCK, BMAL1, PER1/2/3, and CRY1/2), glucose, and lipid metabolism-related genes? *Hepatic cell biology in vitro systems*: Do hepatic cell biology systems (liver slices, primary cells, cell lines) provide a viable experimental approach to assess circadian rhythmicity in diabetes?

A keyword search was conducted by using search terms for humans (*night shift, glucose insulin, circadian, and misalignment’*), rodents (*circadian, circadian rhythm, diurnal, rodent, metabolism, diabetes, and high-fat*), and cell biology (*circadian rhythm, diurnal, diabetes, and hepatocytes*). We choose to focus on markers of metabolic disease, specifically fasted plasma glucose, insulin, and lipids.

## HUMAN CIRCADIAN STUDIES

Circadian human studies are difficult to undertake due to technical challenges of controlling for the environment and its powerful timing cues (zeitgebers) of light, and food. Equally, reductionist studies in highly artificial environments can make the practical impact of these studies limited (37). Despite these limitations, human studies provide insight into how alterations to the circadian system can impact upon metabolic health. Here, we review studies investigating metabolic impact (glycemic control) of circadian disruption in both real-world shift workers and in otherwise healthy individuals subjected to misalignment protocols. A total of 120 studies were obtained and 22 original studies were kept after screening abstracts and excluding studies that did not meet the inclusion criteria (see Supplemental Fig. S1). Search results are summarized in Table 1 for shift work studies, and Table 2 for misalignment studies.

**Table 1.** Effect of shift work on markers of fasting glucose and insulin and lipid profiles

References	Study Design	Gender	Age	BMI, kg/m^2^	Study Population	Plasma Glucose	Plasma Insulin	Lipid Profile
Rizza et al. (38)	Cross-sectional study	M (62) F (100)	44.1–40.3	24.2–25.9	Day workers (DWs) vs. rotating night shift workers (rNWs)	↔ Fasting glucose (DWs vs. rNWs)	↔ Fasting insulin (DWs vs. rNWs) ↔ Fasting HOMA-IR (DWs vs. rNWs)	↔ TG (DWs vs. rNWs) ↔ Total cholesterol (DWs vs. rNWs) ↔ HDL cholesterol (DWs vs. rNWs) ↔ LDL cholesterol (DWs vs. rNWs)
Ledda et al. (39)	Cross-sectional study	M (133) F (139)	39–41	21–22	Healthcare workers (nSWs) vs. healthcare shift workers (SWs)	↑ Fasting glucose (nSWs vs. SWs)	↑ Fasting insulin (nSWs vs. SWs) ↑ Fasting HOMA-IR (nSWs vs. SWs)	Not measured
Kiranmala et al. (40)	Cross-sectional study	M (26) F (14)	29–30	23.7–24.4	Nonshift workers (nSWs) vs. shift workers (SWs)	↔ Fasting glucose (nSWs vs. SWs)	↔ Fasting insulin (nSWs vs. SWs) ↔ Fasting HOMA-IR (nSWs vs. SWs)	↔ TG (nSWs vs. SWs) ↔ Total cholesterol (nSWs vs. SWs) ↔ HDL cholesterol (nSWs vs. SWs) ↔ LDL cholesterol (nSWs vs. SWs)
Sharma et al. (41)	Randomized crossover study	M (2) F (10)	25 ± 1	26.9 ± 1.0	Day workers (DWs) vs. night workers (NWs) Participants assessed after working two consecutive 12 h shifts (either day or night), during their third 12 h shift period for either day or night shift study	↔ Fasting glucose (DWs vs. NWs)	↓ Fasting insulin (DWs vs. NWs)	Not measured
Schiavo-Cardozo et al. (42)	Cross-sectional study	F (24)	20–40	25–29.9	Day workers (DWs) vs. night workers (NWs) Day shift—business hours Night shift—7:00 PM to 7:00 AM	↔ Fasting glucose (DWs vs. NWs)	↔ Fasting insulin (DWs vs. NWs) ↔ HOMA-IR (DWs vs. NWs)	↑ TG (DWs vs. NWs) ↔ Total cholesterol (DWs vs. NWs) ↔ HDL cholesterol (DWs vs. NWs) ↔ LDL cholesterol (DWs vs. NWs)
Padilha et al. (43)	Cross-sectional study	M (21)	25–35	NS	Day workers (DWs), early morning workers (EMWs), night workers (NWs)	Not measured	↔ HOMA-IR	Not measured
Wehrens et al. (44)	Cross-sectional study	NS	25–45	21.5–35.7	Non shift workers (nSWs) vs. shift workers (SWs)	↔ Fasting glucose (nSWs vs. SWs)	↔ Fasting insulin (nSWs vs. SWs)	↔ TAG (nSWs vs. SWs) ↔ NEFAs (nSWs vs. SWs)
Lund et al. (45)	Cross-sectional study	M (10) F (2)	24–34	NS	Shift workers: 7-day shifts: 9:00 AM to 5:00 PM (DWs) 7-night shifts: 12:00 AM to 8:00 AM (NWs)Antarctic survey station	↔ Fasting glucose (DWs vs. NWs) ↑ Postprandial glucose in after 9 days of shift work-improved when switched back to day shifts	↔ Fasting Insulin (DWs vs. NWs)	↔ NEFAs (DWs vs. SWs) ↑ Postprandial TAG and NEFA on the second day of night-shift work- remained elevated 2 days following the return to daytime working

Values in parentheses in Gender column represent number of subjects. HOMA-IR, homeostatic model assessment for insulin resistance; NEFA, nonesterified fatty acids; TAG, triacylglycerol; TG, triglycerides.

**Table 2.** Human misalignment studies measuring fasting glucose, insulin, and lipid profiles

References	Study Design	Gender	Age	BMI, kg/m^2^	Misalignment Protocol	Plasma Glucose	Plasma Insulin	Lipid Profile
*Simulated night shift work/Circadian misalignment*								
Grant et al. (46)	Control parallel study	11 (M)	18–45	22–23	Simulated night work; 1600–1000 Randomly assigned into eating at night (NE) vs. not eating at night (NEN) NE; *n* = 4 meals; 0700, 1900, 0130 NEN; *n* = 7 meals; 0700, 0930, 1610, 1900	↔ Fasting glucose (NEN vs. NE) Postprandial glucose impaired	↔ Fasting insulin (NEN vs. NE) Postprandial insulin impaired	Not measured
Morris et al. 2016. (10)	Crossover design	M (3) F (6)	24–48	R: 19.3–29.3	3 days *day 1*- sleep/wake cycle inverted by 12 h, followed by staying awake for 16 h Next sleep opportunity- 11:00 AM until 7:00 PM. Sleep/wake protocol maintained until day 3	↔ Fasting glucose (aligned vs. misaligned) Postprandial glucose impaired	↔ Fasting insulin (aligned vs. misaligned) Late phase postprandial insulin 10% higher in circadian misalignment group	Not measured
Morris et al. (47)	Within-participant crossover design	M (8) F (6)	20–49	R: 21–29.5 25.4 ± 2.6	8-day long protocol: Circadian alignment—participants sleep opportunity occurred between 11:00 PM and 7:00 AM. Circadian misalignment—*day 1*–*3* = 11:00 PM–7:00 AM. *day 4—*behavioral cycles shifted by 12 h until end of protocol.	↔ Fasting glucose (aligned vs. misaligned) Postprandial glucose impaired	↔ Fasting insulin (aligned vs. misaligned) ↔ Fasting ISR (aligned vs. misaligned) ↔ Fasting ISR (AUC) (aligned vs. misaligned) Postprandial insulin impaired	↑ Fasting FFA levels (misaligned vs. aligned) ↔ FFA (AUC) (misaligned vs. aligned) ↔ TG (AUC) (misaligned vs. aligned)
Wefers et al. (48)	Randomized crossover design	M (14)	22.4 ± 2.8	22.3 ± 2.1	3-day control protocol and 3.5 day misalignment protocol (12-h rapid shift of cycle). Misalignment participants had matched study procedures to control (e.g., meal time) but 12 h shifted.	↑ Fasting glucose (misalignment vs. aligned)	↑ Fasting insulin (misalignment vs. aligned) ↔ Hepatic insulin sensitivity (misalignment vs. aligned)	↑ Fasting FFA levels (misalignment vs. aligned) ↓ TG (misaligned vs. aligned) ↓ TG (misalignment vs. aligned)
*Dim light vs. bright light at night*								
Albreiki et al. (49)	Randomized two-way crossover study—healthy subjects	M (9) F (8)	22.3 ± 3.6–22.6 ± 2.2	22.9 ± 2.5–22.7 ± 2.2	Dim light (<5 lux) (DL) vs. bright light (BL) (>500 lux)—between 1800 and 0600 the next day	↔ Fasting glucose (DL vs. BL) ↑ Postprandial glucose in bright light group	↔ Fasting insulin (TAUC) (DL vs. BL)	↑ NEFA in dim light (TAUC) ↔ TAGs (DL vs. BL)
*Phase advanced/delay misalignment*								
Gonnissen et al. (50)	Randomized, single-blinded, crossover design	M (7) F (6)	24.3 ± 2.5	23.6 ± 1.7	Phase delay: three light-entrained circadian cycles (3 × 21 h—phase advance or 3 × 27 h—phase delay)	Not measured	↑ Fasting insulin levels (phase delay effect) ↑ HOMA-IR index (phase delay effect)	Not measured
Gonnissen et al. (51)	Randomized single-blinded crossover study	(NS)	24.3 ± 2.5	23.6 ± 1.7	Three conditions—21, 24, and 27 h cycles. Patients participated in the 24-h control cycle and then 21 and 27 cycle conditions were followed in random order. 24 h—subjects slept for 8 h and awoke for 16 h 21 and 27—subjects stayed time blinded in a respiration chamber during 3 light entrained circadian cycles.	↔ Fasting glucose (phase advanced) ↑ Fasting glucose (phase delayed)	↑ Fasting Insulin (phase advanced) ↔ Fasting Insulin (phase delay)	Not measured
Wehrens et al. (44)		NS	25–45	21.5–35.7	Phase delay for 4 days: Awake for 30.5 h followed by a 4-h recovery nap and a recovery sleep	↔ Fasting glucose (baseline vs. sleep deprivation)	↔ Fasting Insulin (baseline vs. sleep deprivation)	↑ Fasting FFA levels (baseline vs. sleep deprivation) ↓ TG (baseline vs. sleep deprivation)
*Sleep fragmentation*								
Stamatakis et al. (52)		M (9) F (2)	18–29	24.3 ± 0.9	Sleep was experimentally fragmented for 2 nights using auditory and mechanical stimuli	↓ Glucose effectiveness (post-fragmentation vs. pre-fragmentation)	↓ Insulin sensitivity (IVGTT) (post-fragmentation vs. pre-fragmentation)	Not measured
*Sleep restriction*								
Eckel et al. (53)	Crossover counterbalanced design.	F (8) M (8)	22.4 ± 4.8	22.9 ± 2.4	Restricted sleep schedule: Simulated 5-day work week of 5 h per night sleep	Not measured	↓ Insulin sensitivity(OGTT and IVGTT)	Not measured
*Sleep restriction with different light exposures*								
Gil-Lozano et al. (54)	Randomized study design.	M (8)	21.1 ± 0.9	23.9 ± 1	T1—normal light-dark cycle with regular sleep T2—sleep deprivation in the dark T3—sleep deprivation with nocturnal light exposure T4—sleep deprivation with nocturnal filtered light exposure	Not measured	↓ Fasting insulin (5 h postmeal) (regular sleep vs. sleep deprived 6:00 AM)	Not measured
*Sleep restriction plus phase delay*								
Buxton et al Shea. (55)		F (10) M (11)	23 ± 2 = young 60 ± 5 = older	(NS)	Spent >5 wk in controlled laboratory conditions. Initial baseline segment of optimal sleep and 3 wk of sleep restriction (5.6 h per 24 h) combined with circadian disruption (recurring 28 h days).	↔ Fasting glucose (baseline vs. sleep deprivation) ↑ Fasting glucose AUC (baseline vs. sleep deprivation)	↓ Fasting insulin ↓ Fasting insulin AUC (baseline vs. sleep deprivation)	Not measured
*Sleep restriction plus circadian misalignment vs. sleep restriction*								
Leproult et al. (56)	Parallel group design	F (7) M (19)	Mean—22.5	Alignment group—23.1 ± 2.4 Misalignment—22.2 ± 2.5	Circadian alignment: 3 inpatient days with 10 h bedtimes followed by 8 inpatient days of sleep restriction to 5 h with fixed nocturnal bedtimes Circadian misalignment: sleep delayed by 8.5 h with sleep restriction to 5 h	↓ Glucose (circadian alignment vs. misalignment)	↑ Insulin (circadian alignment vs. misalignment)	Not measured

Values in parentheses in Gender column represent number of subjects. AUC, area under the curve; FFA, free fatty acid; HOMA-IR, homeostatic model assessment for insulin resistance; ISR, insulin secretion rate; IVGTT, intravenous glucose tolerance test; NEFA, nonesterified fatty acids; OGTT, oral glucose tolerance test; TAG, triacylglycerol; TAUC, time area under curve; TG, triglycerides.

### Metabolic Effects of Shift Work in Humans

Shift work is the most prevalent cause of circadian misalignment, with up to 20% of workers engaged in working shifts, which has been associated with a 10%–40% increased risk of developing T2D (57). Although shift work is associated with other at-risk behaviors including smoking, dietary choices, and sleep deprivation, even after controlling for these factors there is an increased risk of obesity (58, 59). A study comparing healthcare, nonshift workers (HCNSWs) and healthcare shift workers (HCSWs) younger than 50 yr of age with a body mass index (BMI) less than 24.9 kg/m^2^ reported an increase in fasting blood glucose concentration in HCSW compared with HCNSWs (39) (see Table 1). In contrast, another study reported that young (29–30 yr), healthy individuals who had a relatively short duration of shift work (1 yr, 1–2 night shift duties/week) showed no change in fasting plasma glucose and insulin concentrations (40). It has been suggested a minimum of 5 years of shift work is needed for the effects on lipids and glucose metabolism to manifest fully (60, 61). However, other studies have reported that subjects who had worked shift work for at least 5 years showed no difference in plasma fasting glucose, insulin, TG, and nonesterified fatty acids (NEFAs) concentrations when compared with individuals who were not shift workers (44). Therefore, the duration of time it takes for shift work to have a negative impact on plasma markers of glucose, insulin, and lipids in healthy individuals remains uncertain, and it is plausible that effects would be seen earlier in individuals defined as metabolically unhealthy (e.g., increased BMI and hepatic steatosis).

As considering the length of time individuals have been engaged in shift work is an important experimental variable when designing shift worker studies, one study presented in Table 2 provided insight into the metabolic derangement at different time points after shift work (45). They assessed individuals at three time points: *1*) during the daytime of the normal working week; *2*) within 2 wk of commencing the night shift; and *3*) *day 16*, during the daytime on the second day of a return to day working. Although no data were shown, the authors stated that fasting and postprandial plasma glucose, insulin, and NEFAs concentrations were unchanged between day workers and shift workers. They also reported that after working 9 days of shift work there was an increase in postprandial, but not fasting, glucose concentrations, which improved when the subjects switched back to day shifts. In contrast, postprandial TG and NEFA increased on the second day of the night shift work and remain elevated 2 days following the return to daytime working. The observed differences in the responses of plasma glucose and lipids after shift work may be due the dynamics of plasma glucose versus lipids. After food consumption, plasma glucose peaks at 15–30 min after food consumption and returns to baseline after ∼1 h. Whereas, dietary fat has a more prolonged postprandial excursion than glucose (6–8 h in healthy individuals). Dietary lipids are transported in chylomicrons that deliver TG to various tissues, for example, adipose tissue for storage, muscle, and heart for immediate use (62–64). The observed differences in the responses of plasma glucose and lipids after shiftwork may be due to the dynamics of plasma glucose versus lipids. The duration of shift work that results in changes in fasted plasma markers that reflect increased risk of metabolic disease remains unclear. Five years of shift work is considered to be the period of time where the metabolic effects fully manifest but this is yet to be clearly demonstrated (60, 61). Moreover, it is plausible that the duration required to see metabolic effects is dependent/influenced by other factors including an individual’s phenotype (e.g., obesity, T2D, and dyslipidemia), lifestyle factors (e.g., diet and exercise), and gender and race. Current on-going studies will provide longitudinal data that may provide further insight. Another area that requires clarity is how long it takes shift workers to revert back to normal metabolic health, once they stop shift work. Although some have attempted to address this question (65, 66), it would be of interest to study individuals on a longer-term basis (5 year’s plus) to determine how switching between shift work and nonshift work affects metabolic health, and ultimately determine the rate of circadian adaptation (ability to adapt between day and night shifts) [reviewed extensively elsewhere (67)]. One way in which longitudinal information can be achieved without invasive protocols is the use of wearable devices, that can capture heart rate, sleep, and physical exertion throughout the day and night. Although not identified in the systematic search used in this review, studies that have used such technologies have provided valuable insight into shift workers both during their working shifts and their off days providing comprehensive overview of the effects of shift work (68). The advancement of artificial intelligence methodology has also provided further value to the use of wearable devices in human circadian studies (69).

Altogether the studies reviewed indicate that there is much work to be done to improve experimental methods to characterize the relationship between shift work and metabolic disease. The studies included in this review are in healthy individuals (defined by BMI) and thus the findings cannot be fully extrapolated to populations with preexisting metabolic disease who may have abnormal fasting lipid and glucose parameters at baseline. It is plausible that more notable and subtle changes occur in the postprandial phase before a change in fasting concentrations. An alternative explanation may be that healthy individuals metabolically adapt to changes in working hours (such as seen in shift work) through compensatory mechanisms and it is only when circadian patterns are misaligned that more notable effects are evident. Studies that have investigated the effects of shift work in patients with type 2 diabetes have provided evidence that night-shift work is associated with poorer glycemic control (57, 70–72).

### Metabolic Effects of Misalignment Studies in Humans

Circadian misalignment studies involve manipulating timing of sleep, food intake, and light to shift the circadian clock and allow for the assessment of acute response to manipulations. Table 2 summarizes the variety of experimental approaches used for circadian misalignment. From the studies reviewed here that have described the misalignment protocol used as “simulated night shift work,” one study (1/4; 25%) reported a change in fasting glucose and insulin (Table 2) (48). Utilizing a rapid shift protocol, misalignment resulted in a significant increase in fasting plasma glucose, circulating NEFA, and skeletal muscle insulin resistance compared with control conditions (48). The difference in observations from other studies could be explained by the rapid shift protocol that was used. Several studies reviewed in Table 2 (10, 46, 47, 49) also report impaired postprandial glucose and insulin responses under misaligned conditions. Similar to the shift worker studies it may be that fasting responses are more resilient to the effects of alterations to the body clock compared with postprandial responses. Why alterations to the body clock impair postprandial responses to fasting are difficult to explain in current studies but may be due to individual’s phenotype and the length of time they have been engaged in shift work. In addition, measuring postprandial responses can be challenging as perturbations to the system (i.e., food) protocols can be varied and it can be challenging to utilize mixed nutritional challenges. Therefore, fasting measures are often used as a standardized protocol for metabolic studies.

Although not included in the studies reviewed in Table 2, studies that have utilized plasma proteomics have provided interesting insight into the effects of misalignment on the proteome landscape. It has been found that simulated nightshift work has rapid and wide-reaching impacts on 24-h time-of-day protein patterns. It has been reported that overall, 62 proteins had altered 24-h average levels, and 76 had altered 24-h time of day patterns with 11 proteins overlapping between these two categories, resulting in 127 total proteins being altered during circadian misalignment (73). Plasma metabolomics is another technique that has yielded information on the effects of shift work on key metabolic pathways, tryptophan, glutathione, alanine, glycine, and serine metabolism (74). Adoption of techniques such as plasma metabolomics can provide a detailed overview of how the human plasma proteome is altered due to alterations to the body clock. Ultimately, this level of information will allow for greater precision and knowledge focused on the timing of assessing omics health biomarkers. And all of which will lead to improved understanding of healthy physiology and disease processes associated with shift work.

When exploring the role of biological rhythms in human participants, it is challenging to clearly separate the adverse metabolic implications of misalignment from those derived from disrupted sleep. A consistent finding from studies (52–54) that have manipulated sleep as the misalignment protocol (sleep fragmentation and sleep restriction) is reports of reduced insulin sensitivity (Table 2). These findings alongside the studies where sleep has not necessarily been restricted but moved to another time of day, point to the importance of sleep as an independent risk factor (away from just shifts to the body clock) to metabolic health. This is a key consideration when interpreting the findings of shift worker studies or misalignment protocols designed to mimic shift work conditions. Often studies are completed in controlled experimental settings where the study individuals may still be achieving good quality and quantity of sleep, just at a different time of day, and thus not experiencing the consequences of sleep deprivation. Whereas outside of experimental settings, those working shifts may find it difficult to sleep during the day and therefore sleep deprivation is a likely risk factor for metabolic disease in these individuals. In fact, large UK population-based studies have shown that in 277,000 workers, shift workers report less sleep per 24 h/day than nonshift workers, alongside poorer sleep quality (75). Indeed, one of human studies listed in Table 2 combined circadian misalignment with partial sleep loss and reported reduced metabolic rates and increased plasma glucose concentrations, predisposing these individuals to an increased risk of obesity and diabetes (55).

A key consideration that is now emerging and can help to explain the large variation in tolerance to shift work and represents a “natural experiment” through which to dissect the impact of misalignment is by considering chronotype. Chronotype results from complex gene-environment interactions, which may go some way to determining the metabolic consequences of night shift work. Chronotype is the predisposition to be a morning or evening person and has been reported to have an impact on the metabolic effects of shift work (76). Carriers of a *PER2* missense variant have a longer circadian period measured in controlled experimental conditions (77), and tend to have a late chronotype. It has been suggested that late chronotypes (that is individuals who prefer to wake and sleep later in the day) suffer less from the negative effects of circadian and sleep disruption when working night shifts, as compared with early chronotypes (78, 79). Studies investigating the effect of chronotype in nurses have provided further evidence, as those who reported as intermediate chronotype, but not late chronotype, showed an increased risk of T2D with longer durations of night shift work (79). Therefore, taking into consideration chronotype may help in better identifying acute and chronic health effects of shift work and allow for individually tailored prevention strategies.

Taken together, misalignment studies have provided insight into how alterations to the body clock can result in increased risk of metabolic consequences, in particular postprandial glucose and insulin appear to have a stronger response to alterations to the body clock than fasting responses.

### Lessons from Human Circadian Studies So Far

Shift work and misalignment studies have provided insight into how alterations to the body clock can result in metabolic consequences in healthy individuals (80). The studies reviewed here have focused on fasting plasma glucose, insulin, and lipid parameters. Further expanding experimental techniques such as the use of isotope tracers in human circadian studies to measure gluconeogenesis and de novo lipogenesis will provide detailed mechanistic insight to link circadian mechanisms with metabolic liver disease (81, 82). A further key point to note from the studies reviewed in Tables 1 and 2 and simulated shift work studies not reviewed here is that the majority are conducted predominately in male participants. Sexual dimorphism in many biological processes and diseases is now well established (83, 84). Recent studies are providing evidence of a link between sexual dimorphism and circadian molecular machinery in humans (85). In addition, although outside the scope of this paper, the role of race in metabolic circadian oscillations in humans is an area that requires further investigation. For a comprehensive overview of the role of race, sex, and age in circadian metabolism see the work by Zhang et al. (86).

There remain many unanswered questions in relation to the metabolic effects of shift work and circadian misalignment, most likely due to the heterogeneous nature of shift work schedules and misalignment protocols used. The current limitations and methodological considerations in human shift worker studies are reviewed elsewhere (87, 88). One potential interesting area that requires further exploration beyond just profiling the metabolic effects of shift work is strategies to realign the body clock that could be utilized by shift workers. Time-restricted feeding, defined as restricting food intake to 8–12 h each day (89), has been shown in human studies to reduce body weight (90), and improvements in glucose and LDL and HDL cholesterol (91, 92).

Human circadian studies have furthered our understanding of how alterations to the body clock, through shift work or misalignment, can impact upon metabolism to potentially increase the risk of metabolic diseases including T2D. However, due to the limitations associated with human studies evidence of the role the specific endocrine organs play in metabolic disturbances remains to be fully elucidated. Rodent studies can go some way to addressing this knowledge gap and will be the focus of the next section.

## RODENT CIRCADIAN STUDIES

To better assess how circadian regulation of metabolism relates to metabolic disturbances, mouse models of obesity have been instrumental. Animal models help bridge the gap between human and in vitro studies allowing for organ-specific changes to be assessed, and the possibility of genetic and dietary, and activity-related manipulations. For this systematic search, we chose to focus on the liver due to the key metabolic role it plays in regulation of glucose and lipid metabolism. A total of 168 studies were obtained and 12 original studies were kept after screening abstracts and excluding studies that did not meet the inclusion criteria (see Supplemental Fig. S2). Search results are summarized in Table 3.

**Table 3.** Hepatic circadian, glucose and lipid genes in dietary rodent models of obesity

References	Species	Dietary Intervention	Phenotype	Effect of Diabetic Intervention on Expression of Hepatic Circadian Genes across Time	Effect of Diabetic Intervention on Expression of Liver Glucose Metabolism Genes across Time	Effect of Diabetic Intervention on Expression of Lipid Metabolism Genes across Time
			(changes in blood glucose, body weight)	*arrow indicates change from control sample	*arrow indicates change from control sample	*arrow indicates change from control sample
		Dietary models				
Oosterman et al. (93)	Rat (Wistar, male)	5 wk—Saturated fat (beef tallow) and 30% sucrose water	↑ WAT ↑ Hepatocytes with fat ↑ Plasma insulin	↔ *Per2* (ZT0–ZT24) ↔ *Bmal1* (ZT0–ZT24) ↔ *Cry1* (ZT0–ZT24) ↔ *Rev-erbα* (ZT0–ZT24)	↑ *Pgc1α* at ZT12 ↑ *Glucokinase* at ZT12 *No statistics provided	↑ *Acc* at ZT24 ↔ Fas *No statistics provided
Woodie et al. (94)	Mouse (C5Bl/6N, male)	16 wk HFD (40% fat)	↑ Body weight↑ GTT ↑ Blood glucose (ZT5) ↑ Serum insulin (ZT13, ZT17, and ZT21)	↓ *Bmal1* (ZT17 and ZT21) ↓ *Rev-erbα* (ZT9 and ZT13) ↔ *Clock* ↔ *Per2* ↔ *Cry1*	Not measured	Not measured
Quagliarini et al. (95)	Mouse (C57BL/6J, male)	12 wk HFD (58% fat with sucrose)	↑ Body weight ↑ Fat mass ↓ Lean mass	↔ *Cry1* (ZT0–ZT20) ↔ *Cry2* (ZT0–ZT20) ↔ *Per1* (ZT0–ZT20) ↔ *Per2* (ZT0–ZT20) ↔ *Bmal1* (ZT0–ZT20) ↔ *Rorα* (ZT0–ZT20) ↔ *Rorγ* (ZT0–ZT20) ↔ *Rev-erbα* (ZT0–ZT20) ↔ *Rev-erbβ* (ZT0–ZT20)	RNA-Seq pathway annotation: ↓ glucose metabolism (ZT0, ZT4, ZT8)	RNA-Seq pathway annotation: ↑ Metabolism of lipids and lipoproteins (ZT0–ZT20) ↑ Fatty acid, triacylglycerol, and ketone body metabolism (ZT0–ZT20)
Toledo et al. (96)	Mouse (C57BL/6J)	8 wk HFD (60% fat)	↑ Blood glucose	↓ *Cry1* (3:00 AM, 7:00 AM) ↓ *Bmal1* (7:00 AM, 11:00 AM) ↑ *Clock* (3:00 AM) ↔ *Per1* (7:00–3:00 AM)	↑ *G6p* (7:00 PM) ↑ *Pck1* (11:00 AM, 3:00 PM, 7:00 PM) ↑ *Fbp1* (7:00 PM)	Not measured
Guan et al. (97)	Mouse (C57BL/6J, male)	12 wk HFD (60% fat)	Not profiled	↔ *Rev-erbα* ↔ *Bmal1* ↔ *Rory* ↔ *Cry1* ↔ *Per2*	Not measured	↑ *Fasn* (enhanced rhythmicity) ↑ *Acaca* (enhanced rhythmicity) ↑ *Acly* (enhanced rhythmicity) ↑ *Elovl5* (enhanced rhythmicity)
Prasai et al. (98)	Mouse (C57BL/6J, male)	10 wk HFD (60% fat)	↑ Body weight ↑ Plasma insulin (2:00 PM and 8:00 PM)	↔ *Bmal1* ↔ *Per2*	↑ *Pepck* at 2 pm	↔ *Ppar*
Eckel-Mahan et al. (29)	Mouse (C57/BL6J, male)	10 wk HFD (60% fat)	↑ Body weight ↑ Liver weight ↑ Blood glucose	↑ *Cry1* at ZT16 ↑ *Per2* (ZT4 and ZT8) ↓ *Per2* (ZT16) ↓ *Bmal1* (ZT4) ↓ *Rev-erbα* at ZT8 ↔ *Clock* at ZT0-ZT24	↑ *Glucokinase* (ZT0, ZT4, ZT8, ZT12, ZT20)	↑ *Pparα* (ZT0–ZT20)
Matsumoto et al. (99)	Mouse (C57/BL6J, male)	7 days HFD (37% fat)	Not profiled	↓ *Rev-erbα* at ZT3	*Srebp1* phase advance	↔ *Dbp*
Sutton et al. (100)	Mouse (C57BL/6J, male)	12 wk HFD (60% fat)	↔ Body weight ↔ Body composition	↔ *Bmal1* ↔ *Per2* ↔ *Rev-erbα*	Not measured	Not measured
Hsieh et al. (101)	Mouse (C57BL/6, male)	11 mo HFD (45% fat)	↑ Body weight ↑ Blood glucose ↑ Plasma cholesterol ↑ Serum insulin	↑ *Per1* at ZT9 ↑ *Per2* at ZT9 ↑ *Per3* at ZT9 ↑ *Cry1* at ZT9 ↑ *Cry2* at ZT9 ↑ *Bmal1* at ZT3 and ZT9 ↔ Clock at ZT3 and ZT9	↑ *Pgc1α* at ZT3 and ZT9 ↔ *Pgc1β* at ZT3 and ZT9 ↑ *Pepck* at ZT3 and ZT9 ↑ *Pdk4* at ZT3 and ZT9	↑ *Dbp* at ZT3
Yanagihara et al. (102)	Mouse (C57/BL6J, female)	8 wk HFD (40%)	↑ Body weight ↑ Blood glucose ↑ Total cholesterol ↑ High-density lipoprotein-cholesterol ↑ Triglyceride	↑ *BMAL1* at ZT18 ↔ *Clock* ↔ *Per1* ↔ *Per2* ↔ *Cry1* ↔ *Cry2*	Not measured	↔ *Dbp*
Kohsaka et al. (103)	Mouse (C57/BL6J, male)	6 wk HFD (60%)		↓ *Clock* at ZT4 and ZT8 ↓ *Bmal1* at ZT4 and ZT8 ↓ *Per2* at ZT16 and ZT20	↑ *SREBP-1c* at ZT4 and ZT8 (Fig. 4D)	↑ *Acc* at ZT0-ZT24 ↑ *Fas* at ZT0, −4, −8, −12, −16, −20 ↑ *Fabp1* at ZT0–ZT24

*BMAL1*, Arnt-like protein; *CLOCK*, circadian locomotor output cycles kaput; *CRY*, *Cryptochrome 1/2*; GTT, glucose tolerance test; HFD, high-fat diet; *PER*, *Period 1/2/3;* ZT, Zeitgeber time.

One of the key questions in the circadian metabolic field is whether alterations to the liver clock precede metabolic disease or does metabolic disease cause alterations to the liver circadian clock. It is well established that the circadian clock exerts remarkable control of metabolism, but also that the information about metabolic state is transmitted back to the circadian clock. This creates a cross talk between metabolic and circadian genes, which in human studies can be difficult to entangle. Therefore, mouse models provide an experimental platform to explore metabolic cross talk and find answers to unanswered questions regarding circadian control of metabolism. As the focus of this review is on liver metabolism, we choose to focus on changes in circadian, glucose and lipid metabolic genes in rodent dietary models of obesity. We acknowledge that gene expression is not necessarily reflective of protein or functional metabolic changes but for comparison of findings across papers this experimental marker was chosen.

### Hepatic Circadian Transcriptional Changes in Rodent Dietary Models of Obesity

Out of the studies looking at circadian transcriptional changes in response to diet-induced obesity included in this review, five (5/12; 42%) reported no change to circadian gene expression across a 24-h period, despite those studies reporting obesity-related phenotypes (increased body weight and glucose intolerance). This provides some evidence that metabolic disturbances can precede defects in the transcriptional control of the circadian clock, however seven (7/12; 58%) studies reported altered expression of at least one of the circadian genes. Interestingly, there appears to be no specific diet duration or composition that causes changes in circadian gene expression, as a wide range of diet duration (5–16 wk) and macronutrient composition (37%–60% fat) have been utilized. One study that reported no change in circadian gene expression across time in a rat model of obesity [saturated fat (beef tallow) and 30% sucrose water] observed that when food intake was shifted to the light phase in rats, which is typically the fed phase, there was a 12-h shift in circadian genes (93). The negative metabolic effects of shifting food intake to the fasted period of the day are a consistent finding in the rodent literature (14, 104). This is in-line with the human studies referenced in human circadian studies that report the beneficial metabolic effects of time-restricted feeding. These observations suggest that a diet rich in fat and sugar can shift the clock to the wrong time, but that also feeding time can further exacerbate the negative impact of a high-caloric diet on metabolic health.

#### Bmal1 and Clock.

Of the studies that reported changes in circadian gene expression in diet-induced obese rodent models (7/12; 58%) (93, 95, 97, 98, 100), there were varying changes in what circadian genes are either up- or downregulated (see Table 3). *Bmal1* and *Clock* genes are the two genes measured most often (7/7 for *Bmal1*; 6/7 for *Clock*). Interestingly, *Bmal1* was reported to change in more studies than *Clock*, with one study reporting increased expression of *Bmal1* at Zeitgeber time (ZT)3 and ZT9 (101) and another study, ZT18 (102), and four studies reporting downregulated expression at ZT4, ZT18, ZT17, and ZT21 (29, 94, 96, 103). Whereas, four studies reported no change in *Clock* gene expression (29, 94, 101, 102), one with upregulation at ZT15 (96), and one with downregulation at ZT4 and ZT8 (103). The one study that reported upregulation of *Bmal1* gene expression was undertaken in female mice (102), whereas all other studies showing no effect was in male mice, suggesting sexual dimorphism in circadian gene nutrient response. Similar to human studies there is an underrepresentation of female animals in circadian-based rodent studies. Sexual dimorphism exists in rodent models in relation to rate of weight gain, metabolic parameters and insulin sensitivity, and expression of key circadian genes. Female rodents have been reported to have the ability to adapt their circadian behavior more rapidly than males (105–107). This would indicate that females may be more protected than males from the negative effects of circadian disruption, such as weight gain. Therefore, the importance of sex differences in chronobiology should not be underestimated.

One explanation for why there is no clear finding on what happens to *Bmal1* and *Clock* gene expression in a rodent model challenged with a high-fat diet is reports that show a nutritional challenge disrupts the *Bmal1 Clock* chromatin recruitment to Nampt resulting in reduced NAD+ (29). Indicating that how the circadian machinery interacts with metabolic end points during a metabolic challenge is more than just alterations to circadian gene expression.

#### Per2 and Cry1/2.

*Period* (*Per2*) and *Cryptochrome* genes make up the negative part of the circadian molecular machinery. Out of the studies reviewed here, *Per2* and *Cry1/2* were not measured in all studies. *Per2* was measured in five studies (5/12; 42%), with two reporting upregulation of *Per2* at ZT4, ZT8, and ZT9, and a further two studies reporting downregulation at ZT16 and ZT20. *Cry1* was measured in five studies (5/12; 42%), with two reporting no change, two showing upregulation at ZT9 and ZT16, and one downregulation at ZT15 and ZT19. In addition to gene expression, it has been reported that protein levels of CRY1 are downregulated at ZT12 in both nucleus and cytosolic fractions, which does not match the gene expression data, indicating the importance of matching up gene expression data with protein measurements (102). *Cry1* has been reported to exert metabolic affects independent of changes in their own gene expression. AMPK has been shown to rhythmically regulate CRY1 stability in mouse livers (108). Fasting glucose and insulin responses are typically impaired in mouse models of obesity, which can lead to inhibition of AMPK, resulting in the inability of AMPK to stabilize CRY1, and consequently metabolic effects on the circadian clock. Indeed, *Cry1* has been linked with regulation of hepatic gluconeogenesis, providing further links between cryptochromes and glucose homeostasis (22).

In contrast to *Cry1*, *Cry2* was relatively underreported in the studies presented in Table 3. One study reported no change in *Cry2* (102) with a second study reporting upregulation at ZT9 (101). The difference in findings between the two studies may be due to diet duration as the study reporting upregulation of *Cry2* fed mice a high-fat diet for 11 mo, and the study with no change fed mice for 8 wk. These findings indicate that *Cry2* is altered at the later stages of metabolic disease; however more studies are required to confirm this. The lack of studies reporting on *Cry2* expression is surprising given that human genome-wide association studies have reported *Cry2* single-nucleotide polymorphisms (SNPs) to be associated with fasting glucose and insulin-related traits (109), and more recent reports showing a significant correlation between hepatic *Cry2* gene expression and hepatic triglyceride content (110). To gain a comprehensive understanding the role of Cry2 in metabolic disease, more studies are required.

#### REV-ERBs and RORα/γ.

The nuclear receptor Reverbα was reported in two studies, and RORα/γ was not reported in any of the studies (see Table 3). The two studies that included observations in Reverbα, reported downregulation at ZT3 and ZT8. Interestingly, one of the studies reported changes after just 7 days of high-fat diet (37% saturated fat). Nuclear receptors are lipid sensors and act as bridge between lipid signaling molecules and transcription responses, and thus are highly responsive to lipid challenges. REV-ERBα has been reported to be a repressor of lipogenic gene expression during the light phase of the diurnal cycle (ZT10) (111). More recent studies have built on the role of REV-ERBα in hepatic lipogenesis and reported that under basal conditions REV-ERBα does not suppress hepatic lipogenesis, but only when metabolically challenged, for example high-fat diet or mistimed feeding (112). Therefore, as the studies reviewed in Table 3 are from mice challenged with a high-fat diet, the observed increase in REV-ERBα indicates REV-ERBα is no longer able to repress lipogenic gene expression to the same extent as control animals and may play an integral role in increased adiposity in high-fat diet mice. The lack of reports identified in this search on RORα/γ is surprising given the established links between ROR and metabolic liver disease. RORα is decreased in liver biopsies taken from patients with NAFLD, and liver-specific RORα knockout mouse models exhibit exacerbated weight gain and insulin resistance. This is mediated by enhanced transcriptional activity of PPARγ resulting in uncontrolled lipogenesis. Although RORα/γ observations are not included in the papers identified in this search there are a number of papers that have reported on nutritional challenges to ROR and observed that RORγ is increased with high-sugar feeding at ZT16 but decreased with high-fat feeding at ZT16 and ZT20 (113). Surprisingly, RORα was unchanged in both the high-sugar fed and high-fat mice, which is similar to other reports in rats fed a high-sucrose diet (114), however these data are in contrast to the observations in human liver biopsies taken from patients with NAFLD and nonalcoholic steatohepatitis (NASH) (115–117). The discrepancy between findings in RORα gene expression in rodent and human studies not only indicates that is more required to clarify the role or RORα in metabolic liver disease, but also that rodent studies may not always represent the ideal model for mimicking human metabolic disease.

From the studies reviewed here, there is no consistent finding of disruption to the circadian genes in a high-fat diet mouse model of obesity. These findings suggest that a diet-induced obesity does not necessarily remodel the circadian clock molecular machinery but may disrupt the rhythmicity of metabolic pathways as reviewed in the next section.

### Hepatic Glucose and Lipid Metabolic Gene Changes in Rodent Dietary Models of Obesity

Glucose transcriptional factors and genes were reported in 8 of 12 studies, with five studies observing upregulation of *Pgc1α*, *Gck*, *G6p*, *Pck1*, *Fbp1*, and *Pdk4*, and one study reporting downregulation of glucose metabolic genes at ZT0, ZT4, and ZT8 (95). Out of these studies there appears to be no trend in the direction of the circadian genes. For example, *Gck* was upregulated in two studies, with one study showed no change (93), whereas another reported an upregulation of *Cry1* and *Per2*, and downregulation of ZT4, and REV-ERBα at ZT8 (29). The divergence in findings may be due to species as one study was in rats and the other in mice and different diet compositions and feeding time between the two studies. *Gck* is an interesting gene from a circadian perspective as it is rhythmic throughout the day, peaking during the feeding period in rodents. Recent reports have shown that administrating *Gck* activator to obese Zucker rats at the start of the feeding period is more beneficial in improving glycemic control and markers of hepatic inflammation, than dosing animals at the start of the fasted period, or continuously (118). How these findings link in with the circadian clock machinery was not explored in this study, but the findings do highlight the therapeutic value of considering time of day for drug administration. How drugs can be repurposed from a chronotherapy perspective is reviewed elsewhere (119). In addition to *Gck*, *G6p*, and *Pck1*, two key genes involved in hepatic gluconeogenesis were elevated in the mouse model of obesity. One study reported that *G6p* was elevated at 7:00 PM, and *Pck1* at 11:00 AM, 3:00 PM, and 7:00 PM (96). Elevated expression of these key genes is not surprising as it is well established that in rodent metabolic disease models there is excessive hepatic gluconeogenesis resulting in increased glucose production and elevated blood glucose levels (120, 121). In addition, *G6p* is involved in lipogenesis as it encodes the rate-limiting enzyme that mediates the reaction step of NADPH production in the pentose phosphate pathway. Therefore, identifying optimal day of time to target these genes could be beneficial in reducing excessive gluconeogenesis and lipogenesis. Small molecule compounds that target various circadian genes have shown beneficial effect on glucose homeostasis in mouse models of obesity. Studies utilizing a CRY stabilizer (KL001) in isolated mouse hepatocytes repress glucagon-dependent induction of *Pck1* and *G6p* (122). In addition, in vivo studies utilizing a ROR agonist, nobiletin, have reported blood glucose lowering effects in rodent models of obesity, but mechanisms of ROR regulation of gluconeogenesis are yet to be determined (123).

In contrast to the studies reporting increased expression of glucose metabolic genes, one study (95) reported a decrease (glucose metabolic genes based on RNA-Seq pathway annotation) at ZT0, ZT4, and ZT8. It is not clear why this finding is different to other studies included in Table 3 as the duration and composition of diet were not drastically different to other studies, and animals had increased body weight and fat mass. Interestingly, lipid metabolism genes were upregulated in this study that is similar to reports in the other studies included in Table 3. Diet-induced obesity was reported in one study to increase the rhythmicity of genes involved in de novo lipogenesis (*Fasn, Acaca, Acly, Elovl5*), and also the rate of hepatic fatty acid synthesis as measured by tracing deuterated water into newly synthesized fatty acids (97). This study also showed increased rhythmicity in fatty acid oxidation genes (*Acox1, Aldh3a2, Acca1, Hadh*), and that this process was upregulated at ZT10 relative to ZT22. This study went on to determine that targeting PPARα with PPAR agonists at the peak of its expression (ZT8) was more beneficial in reducing lipid metabolic genes and serum TG that using the agonist at the trough of PPARα ZT20. Thus, providing further evidence of how considering time of day for administration is a key consideration when determining the efficacy of drugs targeting metabolic pathways.

### What Have We Learned from Rodent Circadian Experimental Studies?

Rodent studies provide an experimental model to look at organ-specific circadian effects in a much more tightly controlled environment compared with human studies. It is apparent from the studies reviewed here that there is no consistent finding as to whether liver circadian genes are upregulated, downregulated, or unchanged in a high-fat diet rodent model. The inconsistency in findings may reflect the utilization of different dietary fat composition and duration the animals were exposed to the diet. It is also important to consider how housing temperature and light-dark cycles may affect findings. There may be different light-dark schedules between laboratories and animal facilities, and potentially unintentional light disruption at night will cause profound disruption to circadian rhythm and thus affect experimental results. In addition, stress-induced housing circumstances such as limited nesting opportunities and lack of socialization will cause an increase in stress hormones (cortisol) that will in turn disrupt the circadian clock and may affect glucose metabolism.

Despite some of the limitations of animal studies outlined here, they do allow for in vivo testing of small molecule compounds that target circadian genes to determine how they can potentially be therapeutically beneficial (123–127). Although not reviewed here, genetic mouse models where circadian genes have been knocked out have also been crucial in dissecting out the link between the circadian clock and metabolism, and are extensively reviewed (21). Rodent studies have vastly expanded our understanding of how circadian biology links with metabolism and allowed for further exploration than is possible in human studies. However, there remains a large gap in translating animal research into the clinic, possibly due to differential metabolic pathways and drug responses between animal and models. These disparities may be even more pronounced in the circadian field as rodents are nocturnal compared with humans that are diurnal. Therefore, there is a great drive to develop human centric cellular models that better recapitulate human physiology.

## CELLULAR CIRCADIAN EXPERIMENTAL SYSTEMS

The use of cell biology in vitro systems in the circadian field has expanded over the past decade due to the high throughput nature and the ability for experimental conditions to be manipulated. Cell models provide an environment to test the effect of varying nutrient challenges to the hepatic circadian clock, drug screening, and genetic manipulations. However, there is debate over whether in vitro cell biology models retain circadian rhythmic properties, leading to on-going challenges to improve in vitro hepatic circadian cell biology models. To follow on from the rodent studies reviewed in the previous section, we chose to focus on studies using hepatic in vitro cell biology systems. A total of 42 studies were obtained and eight original studies were kept after screening abstracts and excluding studies that did not meet the inclusion criteria (see Supplemental Fig. S3). Search results are summarized in Table 3.

### Liver Slices, Primary Mouse Hepatocytes, Cell Lines, and Organoids

The utilization of organ slices for circadian measurements has expanded over the past 20 years with the development of the PER2::LUC reporter mouse that contains a PERIOD2:LUCIFERASE fusion protein that can be used as a real-time reporter of circadian dynamics (128). The first reports of the use of liver slices taken from PER2::LUC mouse showed self-sustained oscillations in luminescence from the mPER2::LUC fusion protein for a 20-day period (128). A reduction in amplitude was evident at *day 5* of recording, but a discernable circadian rhythm of luminescence in liver was maintained until *day 20*. The use of liver slices from PER2::LUC reporter mice has expanded over the past decade, however, only one study was identified in our search (129). In addition to liver slices taken from PER2::LUC mice, a number of studies included in Table 4 and elsewhere in the literature have isolated primary hepatocytes from the PER2::LUC mouse strain and completed bioluminescence recordings. The advantage of the bioluminescent approach is that it allows for cells to be prepared and cultured in tissue culture plates that can then be run on a luminescence plate reader for multiple days. However, the read out only provides information on Per2 activity and although circadian information can be inferred from this, it only provides data on one circadian gene. Other approaches include the use primary mouse hepatocytes and cell lines where samples are taken for gene expression or protein at numerous time points throughout 24–48 h, as shown in Table 4.

**Table 4.** Cell biology in vitro hepatic systems measuring circadian rhythmicity

References	Cell/Tissue Type	Circadian Experimental Measure	Synchronization Method (Dose, Treatment Time)	ZT Time Period Used (Data shown in paper)	Analysis Approach
*Tissue slices*					
Wible et al. (129)	Liver slices from *Nrf2-*NULL PER2::LUC mouse (MYM and YMK strains)	Bioluminescence of PER2-drive luciferase expression	Dexamethasone (200 nM)	0–5 days	Data recordings excluded if bioluminescence trace did not fit a sine with >80% goodness of fit to the baseline
*Primary cells*					
Guan et al. (130)	Hepatocytes isolated from HepDKO mice	mRNA (*BMAL1, Clock, Cry1, Cry2*, REV-ERBα, REV-ERBβ)	None	0, 4, 8, 12, 16, 20, 24	JKT cycle
Marbach-Breitruck et al. (131)	Hepatocytes isolated from PER2::LUC mice	Bioluminescence of PER2::luciferase expression	None	0–180 h	Chronostar software
Jacobi et al. (132)	Cultured hepatocytes isolated from LBmal1KO mice	Bioenergetic assay at ZT6 and ZT18	Not stated	6, 18 h	Not stated
Liu et al. (133)	Cultured hepatocytes from LLPARDKO mice	Western blot (BMAL1)	Dexamethasone (100 nM, 1 h)	5–35 h	Not stated
Wang et al. (28)	Cultured hepatocytes isolated from *Lgr4*^m/m^ mice	Western blot (BMALl1)	50% horse serum (2 h)	0, 4, 8, 12, 16, 20, 24, 28, 32, 36, 40, 44, 48 h	Not stated for Bmal1 (JKT analysis applied for other genes of interest)
Torra et al. (134)	Cultured hepatocytes isolated from Sprague–Dawley rats	Western blot (REV-ERBα)	Dexamethasone (6 h)	10, 16, 22, 28, 34, 40, 46, 52	Mann–Whitney test comparing levels at the lowest time point (22 h)
*Cell lines*					
Dufour et al. (135)	HepG2	mRNA (*BMAL1, CLOCK, CRY1, CRY2, Per1*, REV-ERBα, REV-ERBβ)	50% horse serum (2 h)	0, 4, 8, 12, 16, 20, 24	Students paired *t* test at each time point
Tracey et al. (136)	HepG2	mRNA (Nr1dp1)	50% horse serum (2 h)	1, 6, 11, 16, 21, 26	Not stated
Lin et al. (137)	HL-7702 hepatocytes (immortalized human cell)	Western blot (PER2, REV-ERBα,)	Dexamethasone (100 nM, 2 h)	24, 28, 32, 36, 40, 44, 48 h	Not stated
Wible et al. (129)	MMH-D3 hepatocytes	Bioluminescence of PER2::luciferase expression Western blot (PER2)	Dexamethasone (200 nM)	0, 4, 8, 12, 16, 20, 24, 28, 32, 36, 40, 44, 48 h	Data recordings excluded if bioluminescence trace did not fit a sine with >80% goodness of fit to the baseline
*Organoids*					
Leone et al. (138)	Hepanoids isolated from C57BL/6 mice	Western blot (PER2, Bmal1)	50% horse serum	8, 16, 24, 32, 40, 48 h	Not stated

*BMAL1*, Arnt-like protein; *CLOCK*, circadian locomotor output cycles kaput; *CRY*, *Cryptochrome 1/2*; *PER*, *Period 1/2/3*; ZT, Zeitgeber time.

In addition to the use of liver slices and primary mouse hepatocytes, cell lines are often used for circadian studies as they can provide a human hepatic model that can be high throughput. Studies identified here using the established cell lines, HepG2 and HL-7702 hepatocytes, report gene expression and protein as outputs across 24- to 48-h period. In addition, one study used a mouse hepatic cell line, MMH-D3 hepatocytes transduced with Per2 lentiviral luciferase reporter (129). Recent advances in the hepatocyte field are the development of three-dimensional (3-D) models that resemble the structure of in vivo tissue, cell-cell and cell-matrix interactions, and provide an in vivo-like biophysical environment. One study was identified that isolated hepatocytes from C57BL/mice and cultured them into hepanoids (3-D cultures of liver cells) and took samples for protein measurements across a 48-h period (138). The use of 3-D liver cell culture is expanding due to the promise that they offer a more physiological environment compared with two-dimensional (2-D) cultures; how they can be used for circadian based studies remains to be determined. The limited amount of studies using in vitro hepatic models for circadian research from our search indicates that there a number of complexities associated with using these models.

#### In vitro entrainment.

Cells in culture for a prolonged period of time can result in desynchronized circadian rhythms. This desynchrony can be restored experimentally with “Zeitgebers,” which are exogenously administered stimuli, for example, serum starvation or dexamethasone for 1–2 h. Common methods used for in vitro synchronization of circadian rhythms have been extensively reviewed (139). Table 4 shows the varying synchronization methods used for the hepatic in vitro cell biology systems identified in this review. Methods used to synchronize the circadian clock in culture may have undesired effects on the in vitro stimulation, in fact, even standard procedures, such as splitting cells or refreshing culture medium can act as synchronizers. Despite this, adding synchronization to a cell culture-based system may improve the physiological relevance of appropriate in vitro methods by reflecting more closely on the biological in vivo situation. It has been reported in human cell line models (HME1, M13SV1, Caco-2, HCT116) that synchronization in vitro can improve cellular response sensitivity in response to chemicals (140), indicating its importance for drug discovery studies.

#### Analysis approaches.

**Figure 2.**
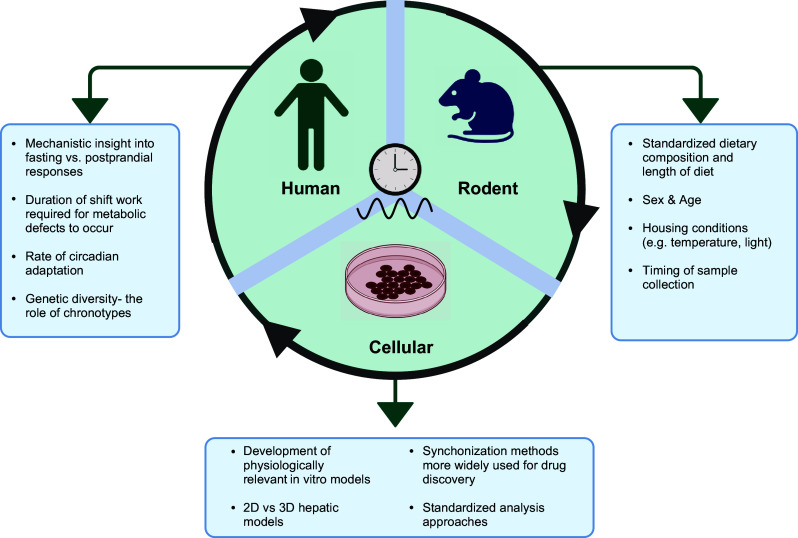
Potential directions for circadian experimental models exploring the role of circadian disruption in metabolism. Figure created using Biorender.com.

Analysis approach needs consideration when interpreting findings from studies using cell biology models for circadian studies. As circadian measurements are made across multiple times points, often spanning 24 to 72 h, data can be noisy, thus statistical modeling, and curve fitting become an important component for accurate interpretation of rhythmic properties. An example of a rhythmic trace and the parameters that can be measured from this are presented in Fig. 2. To analyze this data regression method are often utilized, where the circadian process is represented by a sinusoidal equation (see Fig. 2). Numerous approaches have been previously used to measure rhythms including those based on autocorrelation (141), curve-fitting (142), and Fourier analysis (143). More recent developments in the field have been the introduction of JKT_CYCLE, which is a nonparametric statistical algorithm that can identify and characterize cycling variables in a large dataset (144). The JKT_CYCLE algorithm is available as a computationally efficient R script. Interestingly, only one study identified in this review stated using the JKT_cycle software. There has also been the development of an online platform, Biological Data Repository (BioDare) that allows for data storage, data sharing, and processing and analysis, without the need of R scripts (145).

### Moving Forward with Hepatic In Vitro Cell Biology Circadian Models

An ongoing challenge in circadian biology research is the improvement of current in vitro systems. Current in vitro model systems that have screened small molecule compounds targeting various circadian genes use U2OS PER2:LUC cells and mouse embryonic fibroblasts (MEFS) isolated from PER2::LUC reporter mice (146–148). Both cell models oscillate with high amplitude in culture and thus provide a high throughput platform for testing circadian small molecule compounds; however, neither cell models are hepatocytes. Advancements in imaging techniques such as Circa-SCOPE can provide high-throughput single-cell imaging through time (149), however are yet to be reported to have been used in hepatic cell systems. Therefore, improvement of in vitro hepatic culture systems would allow for more organ- and disease-specific phenotyping, drug screening, and ultimately improve research reproducibility. In vivo animal circadian experiments are a huge undertaking for research teams to capture samples at various time points throughout the 24 h cycle. Even if the animal light-dark cycle is manipulated these studies will still often require sample collection during the night. They also require a large number of animals that highlights how the development of advanced in vitro hepatic circadian models will better comply with the 3R’s (refinement, reproducibility, and reduction).

One area of in vitro development that promises to deliver conditions that better represent the physiological environment is 3-D culture systems, often referred to as organoids. These 3-D structures can be maintained over long periods of time (>1 wk) and allow for complex structures similar to in vivo liver settings as they are able to maintain genetic sustainability. Models such as this can provide high-throughput cell biology for disease modeling, biobanking, drug screening, and toxicity, which go way beyond the maximum capacity of mouse models (150). Studies that have compared primary human hepatocytes (PHH) in 2-D versus 3-D have provided evidence to show that 3-D cultured PHH have higher levels of proteins involved in drug absorption, distribution, metabolism, and excretion, and that the proteome is much more stable compared with 2-D cultured PHH (151). A more extensive review of hepatic 3-D models is provided in Refs. 152 and 153. Organoid models also provide a platform for coculture of different liver cell types (hepatocytes, immune cells, stromal cells, and fibroblasts). This could be a key advancement in the circadian cell biology field as recent reports have shown that Kupffer cells are also influenced by the circadian clock and feeding cues (130). In addition to organoids, hepatocytes cultured in a collagen gel sandwich configuration have been shown to express tight gap junctions and maintain hepatocyte-specific functions (albumin and urea secretion) for several weeks and show robust rhythms compared with hepatocytes culture in 2-D (154). Thus, providing a hepatic cell model that can be used for studies challenging cells with nutritional challenges and drug responses over a period longer than 7 days. In addition, reports using 3-D mammary epithelial cells have provided insight into how stiffening of the mechano-environment can impact circadian function (155). Liver fibrosis is often observed in patients with late-stage NASH (cirrhosis) and thus developing in vitro models that can mimic cell stiffening will be a key advancement in the development of disease-relevant cell biology models (156). Although, the development of 3-D culture systems has significantly advanced over the past decade there are still several limitations including heterogeneity of the size of the organoids, structural and functional issues, and the use of Matrigel that can limit experimental capabilities (150). Despite this, organoid technology does promise to potentially bridge the gap between basic science in vitro work and translational human studies, and thus provide accurate models that can be better predictive tools for chronotherapy and chronopharmacology studies.

## CONCLUSIONS

Over the past few decades, the tools for studying the mechanisms underlying circadian timekeeping and its role in hepatic metabolic regulation have been constantly refined. Challenges remain to bridge the gap between human and rodent circadian studies and improvement in human in vitro models can go some way to providing experimental platforms that can help in generating novel knowledge and therapeutics relating to circadian impact on human health and disease. In addition, although not extensively reviewed here, omics technology such as metabolomics, proteomics, single-cell, and spatial omics have been well adopted in circadian rodent studies (157–159) and are now being used in human and in vitro studies. The utilization of omics technologies will provide far-reaching implications for our understanding of circadian disruption in liver metabolic disease. Further understanding of circadian metabolism is key for better-determining responses to drugs for chronotherapy but can also have important implications for problems such as drug addiction (160) and thus wider societal issues.

The aim of this review was to explore the reasons behind the inconsistent outcomes observed in studies examining the influence of circadian rhythms on metabolism. We have highlighted that background science is not secure, and there is a need for better-designed groups in human and rodent experimental studies, and experimental design to be detailed to adhere to frameworks such as ARRIVE guidelines.

## SUPPLEMENTAL DATA

10.6084/m9.figshare.21750020 Supplemental Figs. S1, S2, and S3: https://doi.org/10.6084/m9.figshare.21750020.

## GRANTS

This work was supported by a Novo Nordisk Postdoctoral Fellowship run in partnership with the University of Oxford (to L.J.D); the British Heart Foundation Fellowship FS/15/56/31645 and FS/SBSRF/21/31013 (to L.H.); Wellcome Trust Clinical Research Training Fellowship Ref. 102176/B/13/Z (to T.M.); DWR MRC Programme Grant MR/P023576/1; and Wellcome Trust 107849/Z/15/Z.

## DISCLOSURES

No conflicts of interest, financial or otherwise, are declared by the authors.

## AUTHOR CONTRIBUTIONS

L.J.D. conceived and designed research; D.K. performed experiments; L.J.D. analyzed data; L.J.D. prepared figures; L.J.D. drafted manuscript; L.J.D., T.M., L.H., and D.W.R. edited and revised manuscript; L.J.D., D.K., T.M., L.H., and D.W.R. approved final version of manuscript.
